# Identification of Genetic and Environmental Factors Suppressing the Lethality and Antibiotic Susceptibility Mediated by Depletion of LptD, a Lipopolysaccharide Transport Protein

**DOI:** 10.4014/jmb.2509.09011

**Published:** 2025-12-09

**Authors:** Umji Choi, Ji Eun Son, Gyubin Han, Chang-Ro Lee

**Affiliations:** Department of Biological Sciences, Myongji University, Yongin, Gyeonggido 17058, Republic of Korea

**Keywords:** Lipopolysaccharide, lipopolysaccharide transport, LptD, suppression, environmental stress, antibiotic resistance

## Abstract

Lipopolysaccharide (LPS), an essential component of the outer membrane (OM) of Gram-negative bacteria, is transported through dedicated Lpt machinery consisting of seven essential proteins. LptD is an OM protein with a β-barrel structure and is involved in the insertion of LPS into the outer leaflet of the OM. In this study, we identified several genetic and environmental factors that partially suppress the lethality and antibiotic susceptibility of the *lptD* mutant. The lethality of the *lptD* mutant was weakened under acidic or salt stress conditions, as well as by the deletion of the *ygfB* gene encoding a hypothetical protein or the *aceE* gene encoding pyruvate dehydrogenase. YgfB physically interacted with the phosphatidylserine synthase PssA, implying that YgfB may be involved in phospholipid biosynthesis. Decreased expression of LptD strongly increased susceptibility to several antibiotics, such as clindamycin and rifampicin, and these increased susceptibilities were partially weakened by the deletion of *aceE* or *ygfB*. Collectively, these results can improve our understanding of the physiological functions and clinical importance of LptD.

## Introduction

The lipopolysaccharide (LPS) present in the outer membrane (OM) of Gram-negative bacteria renders the OM impermeable to extracellular molecules [1]. The saturated acyl chains of LPS block the passage of hydrophilic molecules, whereas the hydrophilic properties of the core and the lipid A regions restrict the passage of hydrophobic molecules [1]. LPS is synthesized in the cytosol and on the outer leaflet of the inner membrane, and then transported to the OM through a dedicated Lpt machine consisting of seven essential proteins (LptABCDEFG)[2]. The Lpt system forms a transenvelope protein bridge from the outer leaflet of the inner membrane to the outer leaflet of the OM [2]. The LptB_2_FGC complex in the inner membrane functions as an ABC transporter to extract LPS from the inner membrane [3]. The periplasmic chaperone LptA forms a transenvelope protein bridge that spans the entire periplasmic region from the inner membrane to the OM through oligomerization and plays a role in the delivery of LPS to the OM [2, 3]. The LptDE translocon in the OM is involved in the insertion of LPS into the outer leaflet of the OM [2].

LptD is an outer membrane protein (OMP) consisting of an N-terminal periplasmic β-jellyroll domain and a C-terminal β-barrel transmembrane domain [2, 4, 5]. LptE, an OM lipoprotein, tightly interacts with LptD and its periplasmic soluble domain is largely inserted into the barrel lumen of LptD, forming a plug-and-barrel structure [2, 5-7]. The N-terminal periplasmic β-jellyroll domain of LptD binds the lipid A region of LPS, and this interaction is retained while the polysaccharide region of LPS travels through the hydrophilic barrel lumen of LptD [2, 8]. LptD is necessary for the insertion of LPS into the outer leaflet of the OM; therefore, it is an essential protein for bacterial growth. The lethality of the *lptD* mutant was partially suppressed by a null mutation in *bamB* [9] and an insertion mutation in *bamA* encoding other OMPs involved in the assembly of the β-barrel proteins [10], but the underlying molecular mechanisms remain unclear.

In this study, we identified several environmental and genetic factors that partially suppress the lethality and antibiotic susceptibility of the *lptD* mutant. Notably, the growth defects in the *lptD* mutant were partially restored under acidic or salt stress conditions. Additionally, inactivation of *ygfB*, which encodes a hypothetical protein, and *aceE*, which encodes pyruvate dehydrogenase, partially suppressed the lethality of the *lptD* mutant. YgfB directly interacts with the phosphatidylserine synthase PssA, indicating that YgfB may be involved in phospholipid biosynthesis. The depletion of YgfB and AceE also partially restored the increased susceptibility of the *lptD* mutant to antibiotics, including clindamycin and rifampicin. Collectively, these results improve our understanding of the physiological functions and clinical importance of LptD.

## Materials and Methods

### Bacterial Strains, Plasmids, and Culture Conditions

All *E. coli* strains and plasmids used in this study are listed in Table S1. All primer sequences used to construct *E. coli* mutant strains and plasmids are listed in Table S2. Bacterial cells were cultured in Luria–Bertani (LB) medium at 37°C, unless mentioned otherwise. Antibiotics, including tetracycline (10 µg/ml), kanamycin (50 µg/ml), ampicillin (100 µg/ml), and chloramphenicol (5 µg/ml), were added to the culture medium when necessary. The bacterial growth under diverse culture conditions was examined using a 10-fold serial dilution spotting assay. The cells were serially 10-fold diluted from 10^8^ to 10^4^ cells/ml, and 2 µl of diluted samples were spotted onto plates. The plates were incubated at 37°C until colonies of the wild-type cells of 10^4^ cells/ml appeared. The bacterial growth was photographed using a digital camera EOS 100D (Canon Inc., Japan).

All *E. coli* mutant strains were constructed using λ red recombinase, as previously reported [11]. Because *lptD* is essential for bacterial growth, deletion of the *lptD* gene was performed in the wild-type MG1655 strain harboring the plasmid pBAD-LptD expressing LptD under the control of an arabinose-inducible promoter. The FRT sequence containing the kanamycin resistance gene was amplified by polymerase chain reaction (PCR) using primers (Table S2) with a 50 bp sequence for homologous recombination and the pKD13 plasmid as a template. After PCR purification, the DNA products were used to transform MG1655 cells harboring the plasmids pKD46 and pBAD-LptD by electroporation. The transformed cells were spread onto LB plates containing kanamycin (50 µg/ml) and arabinose (0.1%). Deletion of *lptD* was confirmed by PCR using another primer set (Table S2). The kanamycin resistance gene was removed using the plasmid pCP20 expressing the FLP recombinase. To minimize physiological changes in the bacteria cells, recombination between FRT sequences by FLP recombinase was performed at 37°C, instead of 42°C, as previously reported [12, 13]. Removal of the kanamycin resistance gene was confirmed by PCR. Additional deletions of *ygfB* and *aceE* were created using the same methods.

To construct the pBAD-LptD plasmid, a DNA fragment encompassing the open reading frame of LptD was amplified by PCR using a forward primer with a synthetic *Eco*RI site (underlined) (5'-CTAGCAGGAGGAATTCATGAAAAAACGTATCCCCAC-3'), and a reverse primer possessing a synthetic *Xba*I site (underlined) (5'-GCAGGTCGACTCTAGAGATACCGAGAAGCAGCGTTT-3') (Table S2). After PCR purification, the PCR product was inserted into the plasmid pBAD24 digested with *Eco*RI and *Xba*I by infusion cloning (Clontech, USA), as reported previously [14]. DNA insertion was confirmed by PCR and sequencing. The plasmids pET28a and pET24a for the overexpression of YgfB and PssA, respectively, were constructed using a similar method, except that *Nde*I and *Bam*HI restriction enzymes were used for plasmid digestion. For the bacterial two-hybrid assay, the plasmids pUT18c and pKT25 expressing YgfB and PssA, respectively, were constructed using a similar method, with the exception of the *Bam*HI and *Eco*RI restriction enzymes used for plasmid digestion.

### Measurement of Minimal Inhibitory Concentrations

The minimal inhibitory concentrations (MICs) of the antibiotics were measured in LB broth plates according to the guidelines provided by the Clinical and Laboratory Standards Institute [15]. *E. coli* cells cultured overnight in LB broth were inoculated into fresh LB medium. When the turbidity of the McFarland standard reached 0.5 (approximately 1.5 × 10^8^ cells/ml), each culture was diluted to a final concentration of 10^7^ cells/ml using fresh LB broth. Diluted samples (10 µl) were spotted onto LB plates containing each antibiotic at final concentrations ranging from 1,024 µg/ml to 7.8 ng/ml in two-fold serial dilutions. After incubation at 37°C for 20 h, the MICs of the antibiotics were determined. The MICs of the antibiotic indicated the lowest concentration at which visible lawn growth of the cell spot was blocked.

### Transposon Mutagenesis Using Mini-Tn5 and Determination of Transposon Insertion Sites

Transposon mutagenesis was performed using the *pir*-dependent transposon delivery vector pRL27 carrying a Tn5 transposase gene and a mini-Tn5 element encoding a kanamycin resistance protein, as reported previously [14, 16]. Plasmid pRL27 was isolated from the DH5αλ*pir* cells harboring the *pir* gene. The isolated pRL27 was used to transform the *lptD* mutant, which did not carry the *pir* gene. In *lptD* mutant cells, the kanamycin resistance gene can only be maintained when a chromosomal insertion of the mini-Tn5 element occurs. Transformed cells were incubated at 37°C in an LB plate containing kanamycin (50 µg/ml). Mutant cells that suppressed the lethality of the *lptD* mutant were isolated. The chromosomal insertion site of mini-Tn5 was determined by PCR using an arbitrary primer (pRL27-SynArb1) consisting of a GGCGGT sequence and a random sequence, and a mini-Tn5 transposon inner primer (pRL27-inner-F1), 5'-GGTTGTAACACTGGCAGAGCATTACG-3' (Table S2), as described previously [13, 14]. After PCR purification, the PCR product was sequenced using another mimi-Tn5 transposon inner primer (pRL27-inner-F2), 5'-ATCAGCAACTTAAATAGCCTCTAAGG-3' (Table S2).

### Purification of His-Tagged YgfB (His-YgfB)

Overnight cultured ER2566 cells harboring the plasmid pET28a-YgfB were inoculated into fresh LB medium. When the optical density at 600 nm (OD_600_) reached approximately 0.5, 1 mM isopropyl-β-D-1-thiogalactopyranoside (IPTG) was added to the culture medium. After overnight culture at 16°C, cells were harvested. The harvested cells were resuspended in buffer A (50 mM Tris-HCl pH 8.0, 300 mM NaCl) and disrupted using a French pressure cell at 8,000 psi through two passages. After centrifugation at 8,000 ×*g* for 20 min at 4°C, the supernatant was loaded onto 100 μl of TALON metal affinity resin (Clontech) equilibrated with buffer A. The proteins bound to the TALON resin were eluted with buffer A containing 200 mM imidazole. Imidazole in the eluted sample was removed by dialysis for 12 h with 50 mM Tris-HCl (pH 7.5) containing 100 mM NaCl. Purified His-YgfB proteins were promptly used for ligand fishing experiments.

### Ligand Fishing Experiment Using Purified His-YgfB as a Bait

Ligand fishing experiments were performed using purified His-YgfB and TALON metal affinity resins, as reported previously [17, 18]. Overnight cultured MG1655 cells were harvested and resuspended in buffer A. Cells were disrupted using a French pressure cell at 8,000 psi through two passages and centrifuged at 8,000 ×*g* for 20 min at 4°C. The supernatant was mixed with purified His-YgfB as bait or with buffer A as control. The mixtures were loaded into 50 μl of TALON resin in a 1.5-ml tube and incubated for 25 min at 4°C. After washing with buffer A three times, the proteins bound to the TALON resin were eluted with buffer A containing 200 mM imidazole. The eluted proteins were analyzed by sodium dodecyl sulfate-polyacrylamide gel electrophoresis (SDS-PAGE) and stained with Coomassie Brilliant Blue.

### Pull-Down Assay

A pull-down assay was performed using cells overexpressing His-YgfB or PssA, as reported previously [19, 20]. Overnight cultured ER2566 cells harboring the plasmid pET28a-YgfB or pET24a-PssA were inoculated into fresh LB medium. When the OD_600_ reached approximately 0.5, 1 mM IPTG was added into culture medium. After overnight culture at 16°C, cells were harvested and resuspended in buffer A. Cells were disrupted using a French pressure cell at 8,000 psi through two passages and were centrifuged at 8,000 ×*g* for 20 min at 4°C. The supernatant was loaded into 50 μl of TALON resin in a 1.5-ml tube and incubated for 15 min at 4°C. After washing three times with buffer A, the proteins bound to the TALON resin were eluted with buffer A containing 200 mM imidazole. The eluted proteins were analyzed by SDS-PAGE and stained with Coomassie Brilliant Blue.

### Bacterial Two-Hybrid Assay

The *in vivo* interaction between YgfB and PssA was assessed using a bacterial two-hybrid assay, as reported previously [14, 17, 21]. The plasmid pUT18c-YgfB expressed a YgfB protein fused with T18 of adenylate cyclase at the N-terminus, whereas the plasmid pKT25-PssA expressed a PssA protein fused with T25 at the N-terminus. The two plasmids were used to transform BTH101 cells lacking adenylate cyclase by electroporation. Transformed BTH101 cells were spotted onto an LB plate containing IPTG (1 mM), ampicillin (100 µg/ml), kanamycin (50 µg/ml), and 40 µg/ml 5-bromo-4-chloro-3-indolyl-β-D-galactopyranoside (X-gal). Zip was designated as the GCN4 leucine zipper moiety and used as a positive control. The color of the cells was measured after incubation for 24 h at 30°C.

## Results

### The Growth of the *lptD* Mutant in LB Medium Is Partially Restored under Acidic or Salt Stress Conditions

**Fig. 1:**
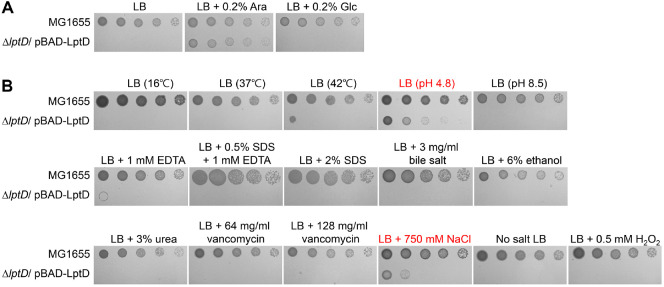
The lethality of the *lptD* mutant was partially suppressed under acidic or salt stress conditions. (**A**) The essentiality of the *lptD* gene. (**B**) The growth of the *lptD* mutant under various stress conditions. (**A** and **B**) The cells of the indicated strains were serially diluted from 10^8^ to 10^4^ cells/ml in 10-fold steps and spotted onto LB plates, LB plates containing indicated materials, or an LB plate adjusted to pH 4.8 or 8.5. No salt LB indicates LB medium without NaCl. All experiments were performed in triplicate, and a representative image is presented.

To better understand the physiological role of LptD, we investigated the environmental stress conditions under which the essentiality of LptD is diminished. We constructed an *lptD* mutant harboring the pBAD-LptD plasmid expressing LptD under an arabinose-inducible promoter. This mutation was lethal in LB medium with or without 0.2% glucose, but it was not lethal in LB medium containing 0.2% arabinose (Fig. 1A), confirming successful construction of the *lptD* mutant. Under most stress conditions, the *lptD* mutantion was lethal; however, under acidic or salt stress conditions, the mutant grew slightly, despite the absence of arabinose (Fig. 1B). These results indicate that the essentiality of LptD is alleviated under acidic or salt stress conditions.

### Deletion of the *ygfB* or *aceE* Gene Partially Suppresses the Lethality of the *lptD* Mutant

**Fig. 2:**
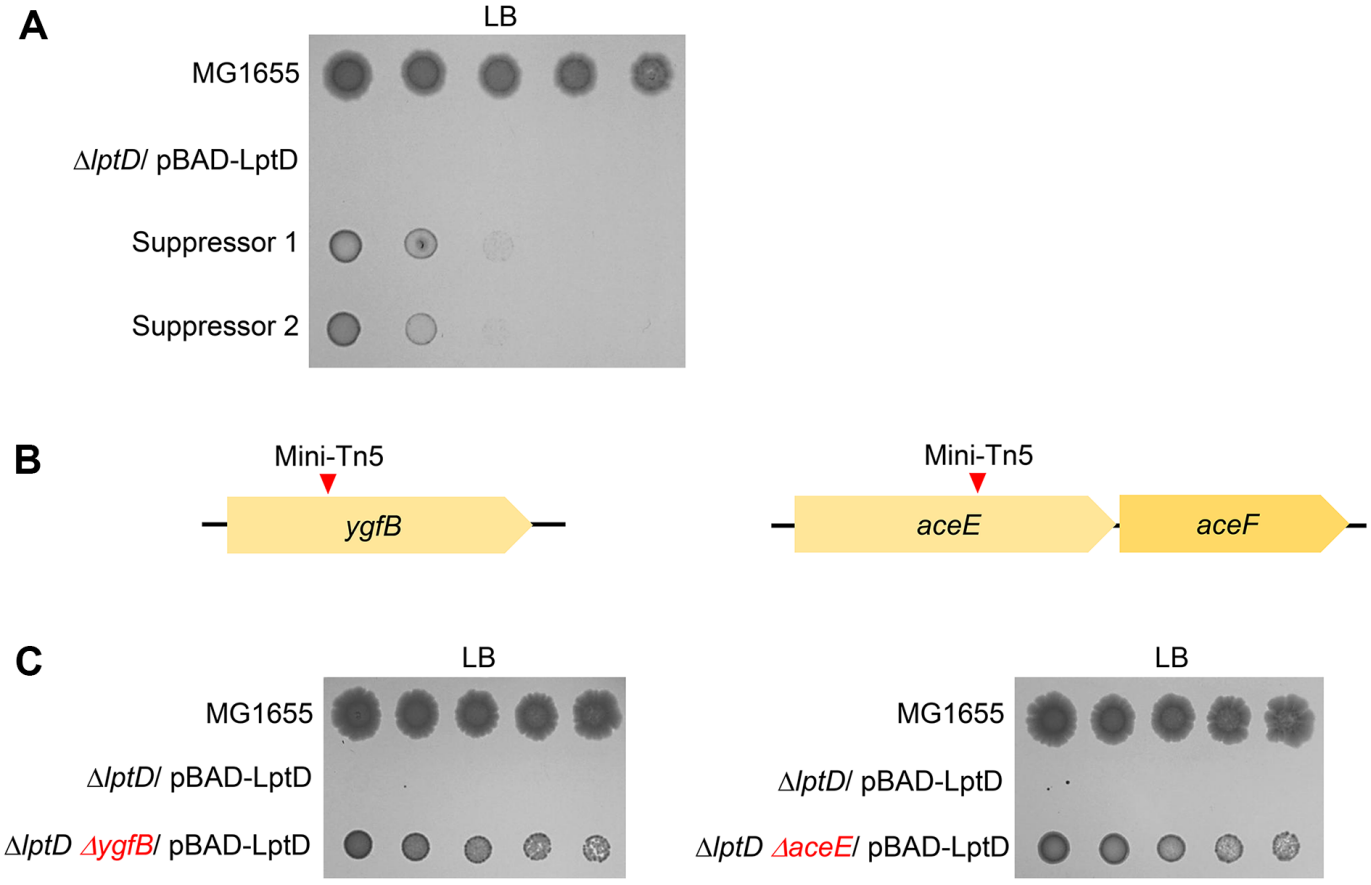
The lethality of the *lptD* mutant was partially suppressed by deletion of *ygfB* or *aceE*. (**A**) Isolation of the suppressor mutant of the *lptD* mutant. The cells of the indicated strains were serially diluted from 10^8^ to 10^4^ cells/ml in 10-fold steps and spotted onto an LB plate. (**B**) Schematic representation of a mini-Tn5 insertion site. The mini-Tn5 insertion sites of suppressor mutants are indicated using red arrows. (**C**) Depletion of *ygfB* or *aceE* partially suppressed the lethality of the *lptD* mutant. The cells of the indicated strains were serially diluted from 10^8^ to 10^4^ cells/ml in 10-fold steps and spotted onto LB plates. The experiments were performed in triplicate, and a representative image is presented.

To identify the genetic factors that suppress the lethality of the *lptD* mutant, we performed random mutagenesis using a mini-Tn5 transposon in LB medium. Two suppressors that partially alleviated the lethality of the *lptD* mutant were identified (Fig. 2A). The transposon insertions were mapped within *ygfB* encoding a PF03695 family hypothetical protein, and *aceE*, which encodes the pyruvate dehydrogenase component (E1) of the pyruvate dehydrogenase complex (Fig. 2B). To confirm the effects of transposon insertion, we constructed two double mutant strains with additional deletions of the *ygfB* or *aceE* gene in the *lptD* mutant. Both the *ygfB* *lptD* and *aceE* *lptD* double mutant strains grew slightly in LB medium without arabinose (Fig. 2C). These results suggest that the inactivation of YgfB or AceE partially suppresses the lethality of the *lptD* mutant.

### YgfB Directly Interacts with Phosphatidylserine Synthase PssA

**Fig. 3:**
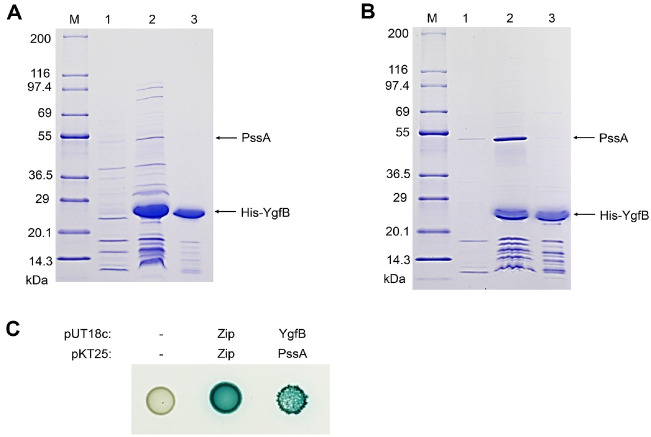
The physical interaction between YgfB and PssA. (**A**) Ligand fishing experiment using His-YgfB as bait. The crude extract from MG1655 grown in 200 ml of LB medium was mixed with buffer A (lane 1) or 50 μg of purified His-YgfB (lane 2). These mixtures were incubated with 50 μl of TALON resin for metal affinity chromatography. The proteins bound to each column were processed as described in Materials and Methods. Purified His-YgfB was loaded as a control (lane 3). EzWayTM Protein Blue MW Marker (KOMABIOTECH) was used as the molecular mass marker (lane M). (**B**) The binding test between YgfB and PssA. Crude extract from ER2566 overexpressing PssA grown in LB medium was mixed with buffer A (lane 1) or crude extract from ER2566 overexpressing His-YgfB grown in LB medium (lane 2). Additionally, crude extract from ER2566 overexpressing His-YgfB grown in LB medium was mixed with buffer A (lane 3). These mixtures were incubated with 50 μl of TALON resin for metal affinity chromatography. The proteins bound to each column were processed as described in Materials and Methods. EzWayTM Protein Blue MW Marker (KOMABIOTECH) was used as the molecular mass marker (lane M). (**C**) The bacterial two-hybrid assay to assess the *in vivo* interaction of YgfB with PssA. The indicated strains were spotted onto LB plates containing 40 µg/ml X-Gal and 1 mM IPTG and incubated at 30°C for 24 h. Zip designates the GCN4 leucine zipper moiety used as positive control.

YgfB is an uncharacterized protein that belongs to the UPF0149 family of proteins with orthologs found in many g-proteobacteria [22]. The structure of a YgfB ortholog in *Haemophilus influenzae* has been published [23]. YgfB consists of seven α-helices and forms a homodimer (PDB ID 1IZM12). To analyze the cellular role of YgfB, we performed a ligand fishing experiment using purified His-tagged YgfB (His-YgfB) as bait. Total soluble protein extracted from MG1655 cells grown in LB medium was mixed with a metal affinity resin in the presence or absence of purified His-YgfB. In several experiments, we identified an interacting protein with a molecular mass of approximately 50 kDa that appeared only in the fraction containing purified His-YgfB (Fig. 3A, lane 2). Through peptide mapping after in-gel tryptic digestion, this protein was identified as phosphatidylserine synthase, PssA, which is involved in the biosynthesis of phosphatidylethanolamine, the most abundant phospholipid in *E. coli* [24, 25]. To confirm the specific interactions between YgfB and PssA, we conducted pulldown experiments using overexpressed PssA and His-YgfB. Significant amounts of PssA were pulled down by His-YgfB (Fig. 3B, lane 2). Furthermore, we examined the *in vivo* interactions between YgfB and PssA using a bacterial two-hybrid assay and found that YgfB interacted with PssA (Fig. 3C). Collectively, these results suggest that YgfB directly interacts with PssA.

### Decreased Levels of LptD Lead to Increased Susceptibility to Several Antibiotics such as Clindamycin and Rifampicin

**Fig. 4:**
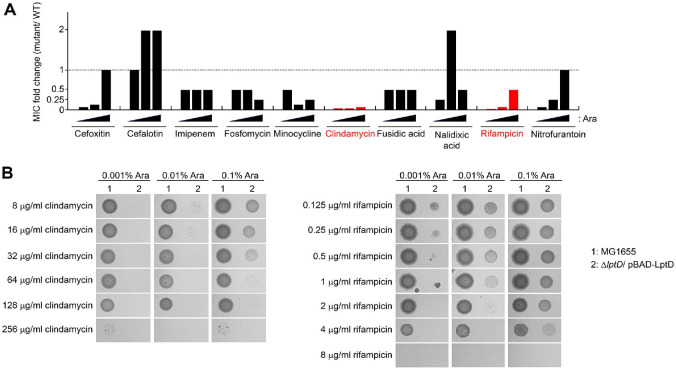
Decreased levels of LptD conferred increased susceptibility to various antibiotics. (**A**) Increased susceptibility of the *lptD* mutant to various antibiotics. The MICs of the indicated antibiotics were measured against the wildtype and *lptD* mutant strains in LB plates containing 0.001%, 0.01%, or 0.1% arabinose (Ara). The relative MIC values for the *lptD* mutant cells in comparison with those for the wild-type cells are presented. (**B**) Significantly increased sensitivity of the *lptD* mutant to clindamycin and rifampicin. The MICs of antibiotics against MG1655 (1) and Δ*lptD* (2) strains were measured in LB broth plates containing the indicated concentrations of antibiotics and arabinose, according to the Clinical Laboratory Standards Institute guidelines.

Since LptD is responsible for the assembly of LPS in the OM, decreased levels of LptD can affect antibiotic resistance. To analyze this hypothesis, we examined the MICs of several antibiotics in the *lptD* mutant harboring the pBAD-LptD plasmid under various concentrations of arabinose (0.001%, 0.01%, and 0.1%) in which the level of LptD decreases. The MIC values of most antibiotics determined by the agar dilution method remained unchanged regardless of the arabinose concentration, whereas those of several antibiotics, including clindamycin, rifampicin, cefoxitin, and nitrofurantoin, decreased in an arabinose concentration-dependent manner (Fig. 4A and Table S3). In particular, the MICs of clindamycin and rifampicin in the *lptD* mutant were 32-fold and 64-fold lower, respectively, than those in the wild-type strain when the concentration of arabinose was 0.001% (Fig. 4B). When the concentrations of arabinose increased, the decrease in the MICs of clindamycin and rifampicin in the *lptD* mutant gradually diminished (Fig. 4B). Therefore, these results indicate that LptD is required for intrinsic resistance to several antibiotics, such as clindamycin and rifampicin.

### The Increased Susceptibility of the *lptD* Mutant to Clindamycin and Rifampicin Is Decreased at the Background of Deletion of *ygfB* or *aceE*

**Fig. 5:**
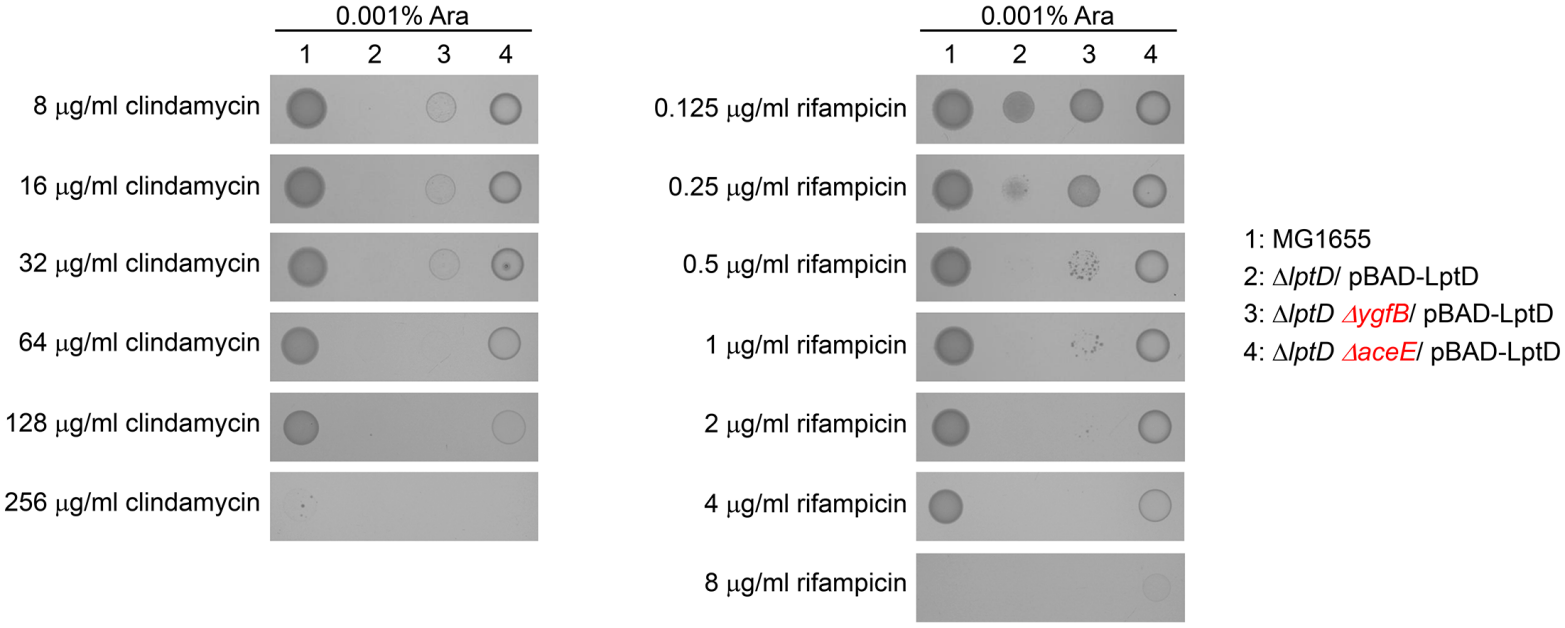
Deletions of *aceE* and *ygfB* partially restored the growth of the *lptD* mutant in the presence of clindamycin or rifampicin. The MICs of the antibiotics against the MG1655 (1), Δ*lptD* (2), Δ*lptD* Δ*ygfB* (3), and Δ*lptD* Δ*aceE* (4) strains were measured in LB broth plates containing the indicated concentrations of antibiotics and 0.001% arabinose (Ara), according to the Clinical Laboratory Standards Institute guidelines.

The deletion of *ygfB* or *aceE* partially suppressed the lethality of the *lptD* mutant (Fig. 2C). We wondered whether the deletion of these genes would also affect the increased susceptibility of the *lptD* mutant to clindamycin and rifampicin. Deletion of *aceE* significantly increased the resistance of the *lptD* mutant to clindamycin and rifampicin, whereas deletion of *ygfB* slightly increased the resistance to these antibiotics (Fig. 5). These results indicate that genetic factors that suppress the lethality of the *lptD* mutant affect the antibiotic resistance associated with LptD.

## Discussion

The transport of LPS into the OM by the Lpt complex is essential for the intracellular functions of LPS [2, 8]. LptD is a component of the Lpt complex, which plays a pivotal role in the insertion of LPS into the outer leaflet of the OM [2, 7]. In the present study, we identified several genetic and environmental factors that suppress the lethality of the *lptD* mutant. Stress conditions, including acidic and salt stresses, partially suppressed the lethality of the *lptD* mutant (Fig. 1). Furthermore, deletions of two genes, *ygfB* and *aceE*, partially restored the growth defects of the *lptD* mutant (Fig. 2). These results provide clues for understanding the LPS-related bacterial physiology.

Several studies have reported that the essentiality of certain proteins disappeared under stress conditions. For example, MepS and MepM, major peptidoglycan endopeptidases, show synthetic lethality in LB medium [26, 27], but not in minimal medium [28]. The cytoskeletal protein RodZ is essential when cells are grown in LB medium at 30°C or below, but not in minimal medium [29]. In this study, we found that the essentiality of LptD partially disappeared under acidic or salt stress conditions (Fig. 1). However, the molecular mechanisms underlying these phenotypes remain unclear.

We identified two genes, *ygfB* and *aceE*, that are involved in the suppression of the growth defects in the *lptD* mutant (Fig. 2). YgfB is a cytosolic protein of unknown function; however, we identified the phosphatidylserine synthase PssA as a binding partner of YgfB (Fig. 3). These results indicate that YgfB is involved in the regulation of phospholipid biosynthesis. Notably, AceE, a component of the pyruvate dehydrogenase complex, also affects phospholipid biosynthesis. The pyruvate dehydrogenase complex catalyzes the conversion of pyruvate into acetyl-CoA, a key substrate for the biosynthesis of fatty acids, which are key building blocks for phospholipid biosynthesis [30]. Therefore, our results suggest that regulation of phospholipid biosynthesis affects the essential role of LptD. Because the OM is an asymmetric membrane composed of phospholipids and LPS, coordinated biosynthesis of the two lipids may be important for proper formation of the OM. Interestingly, recent several reports showed that cardiolipin facilitates the transportation of LPS in the inner membrane into the OM [31, 32]. Additionally, *E. coli* mutants defective in PssA resulted in cardiolipin accumulation in the inner and outer membranes [33]. Therefore, if YgfB might activate the activity of PssA, a plausible mechanistic model can be proposed. Decreased activity of PssA by depletion of YgfB can result in increased levels of cardiolipin, which may enhance the transportation of LPS in the inner membrane into the OM. As deletion of *aceE* can decrease the biosynthesis level of phosphatidylethanolamine through low levels of acetyl-CoA, the same mechanism can be acted upon this case. Similarly, the partial growth restoration of the *lptD* mutant under salt stress can be also explained by the same mechanistic model. Expression of *clsA* was strongly enhanced under high salt conditions [34]. Therefore, salt stress can facilitate the transportation of LPS in the inner membrane into the OM via cardiolipin accumulation. Experiments to prove these suggested mechanistic models can be performed in further studies.

Previous studies have reported that the *lptD* mutant (*imp4213*) is sensitive to many antibiotics such as erythromycin and rifampin [9, 10, 35]. This increased susceptibility was partially suppressed by mutations in *bamB* [9] or *bamA* [10]. Similarly, our study showed that the *lptD* mutant was strongly sensitive to two hydrophobic antibiotics of high molecular weight, clindamycin and rifampicin (Fig. 4), and that these phenotypes were partially suppressed by the deletion of *aceE* or *ygfB* (Fig. 5). These results suggest that LptD is a good target for the development of novel potential adjuvants to clindamycin and rifampicin as well as for the development of novel antimicrobial agents.

A recent study showed that YgfB regulates β-lactam resistance and peptidoglycan turnover via direct interaction with AlpA, an antiterminator protein which controls the expression of the peptidoglycan amidase AmpDh3 [22]. Our study suggests that YgfB might regulate the biosynthesis of phospholipids via direct interaction with PssA (Fig. 3). Therefore, these results imply that YgfB can act as an important regulator controlling the biosynthesis of peptidoglycan and phospholipids. Further studies about this point are required to reveal the physiological roles of YgfB. Additionally, systematic effects of YgfB on antibiotic resistance can be investigated in further studies.

In this study, we identified several novel environmental and genetic factors associated with LptD. Two genes involved in phospholipid biosynthesis were associated with suppression of lethality and antibiotic susceptibility in the *lptD* mutant. These results can improve our understanding of the physiological functions and the clinical importance of LptD.

## Supplemental Materials

Supplementary data for this paper are available on-line only at http://jmb.or.kr.
