# Supplementary material for: Identification of Genetic and Environmental Factors Suppressing the Lethality and Antibiotic Susceptibility Mediated by Depletion of LptD, a Lipopolysaccharide Transport Protein
Source: J Microbiol Biotechnol. 2025 Dec 9;35:e2509011. doi: 10.4014/jmb.2509.09011 (PMC12706146; doi:10.4014/jmb.2509.09011)
Supplement: Supplementary file 1 [file jmb-35-e2509011-supple.pdf]

J. Microbiol. Biotechnol. 2025. 35: e2509011

https://doi.org/10.4014/jmb.2509.09011

Supplementary Tables

Table S1. ***Escherichia coli*** **strains and plasmids used in this study.**

Strain or plasmid	Genotype or phenotype	Source or reference
**Strains**		
MG1655	F^- λ^- *ilvG*$^-$ *rfb*-50 *rph*-1. Wild type *E. coli* K-12	[1]
MG1655 Δ*lptD*	MG1655 *lptD::frt*	This study
MG1655 Δ*lptD* Δ*ygfB*	MG1655 *lptD::frt ygfB::frt*	This study
MG1655 Δ*lptD* Δ*aceE*	MG1655 *lptD::frt aceE::frt*	This study
**Plasmids**		
pBAD24	Expression vector under control of arabinose-inducible promoter, Amp^R	Addgene
pBAD24(Cm)	pBAD24-based expression vector, Cm^R	[2]
pBAD-LptD	pBAD24(Cm)-based expression vector for LptD, Cm^R	This study
pET28a	Expression vector under control of T7 promoter, Km^r	Novagen
pET24a	Expression vector under control of T7 promoter, Km^r	Novagen
pET24a-PssA	pET24a-based expression vector for PssA, Km^r	This study

pET28a-YgfB	pET28a-based expression vector for YgfB with N-terminal 6 histidines, Km^r	This study
pKT25	Cloning vector for creating in frame fusions at the C-terminal end of T25 fragment of *CyaA*, Km^r	[3]
pKT25-PssA	pKNT25(km)-based expression vector for PssA, Km^r	This study
pUT18c	Cloning vector for creating in frame fusions at the C-terminal end of T18 fragment of *CyaA*, Amp^r	[3]
pUT18c-YgfB	pUT18c(amp)-based expression vector for YgfB, Amp^r	This study
pKT25-zip	BACTH control plasmid with the yeast GCN4 leucine zipper fused to the T25 fragment at the C-terminal end, Km^r	[3]
pUT18c-zip	BACTH control plasmid with the yeast GCN4 leucine zipper fused to the T18 fragment at the C-terminal end, Amp^r	[3]
pKD13	Template plasmid for the amplification of the kanamycin-resistance gene bordered by FRT sites, Km^R	[4]
pKD46	λ Red recombinase expression plasmid Ts replicon, Amp^R	[4]
pCP20	FLP helper plasmid Ts replicon, Amp^R, Cm^R	[4]
pRL27	Tn5-RL27 (Km^R-*ori*R6 K) delivery vector: circularized PCR fragment from pRL23 (primers *tet*Ap-for and *oriT-rev*)	[5]

Table S2. Oligonucleotides used in this study.

Name	Oligonucleotide sequence (5’–3’)	Use(s)
LptD-FRT-F	ACCGTTTGTCACGCGCAACGTTACCGATGATGGAACAATAAAATCAACGTGTGTAGGCTGGAGCTGCTTC	Deletion
LptD-FRT-R	ACTGGAACCGTACTTGTTATCGAAATCATTGAAGTAGCTAGGATCGCTGAATTCCGGGGATCCGTCGACC	
YgfB-FRT-F	CCAAAGTGGTAGCATATCATGAATATTCCTCCCTTTGACGACGAATGCTTGTGTAGGCTGGAGCTGCTTC	
YgfB-FRT-R	GCGCGGTCGGTTGCGGATGAGTAAAGGTGTCGTGGCATAACAGCGCGGCAATTCCGGGGATCCGTCGACC	
AceE-FRT-F	GTTTCCCAAATGACGTGGATCCGATCGAAACTCGCGACTGGCTCCAGGCGGTGTAGGCTGGAGCTGCTTC	
AceE-FRT-R	TCAGCGTAGTGATGTAGTAGTAAACGTTCTCTTGTTTTTCACCGTACATAATTCCGGGGATCCGTCGACC	
LptD-cfm-F	GCGTAGAGATGACGAGTACGTTAGTCTCTG	Deletion confirm
LptD-cfm-R	CCATTTCAATTAACCGCACTGCGGATTACG	
YgfB-cfm-F	TATAGCTACCCTGATGAGAAGAGACAAGCC	
YgfB-cfm-R	GGGATATCTCACTCATAACACTCTCCTTAC	
AceE-cfm-F	AGATAGATAAGGAATAACCCATGTCAGAAC	
AceE-cfm-R	GAGCAGCTGAACTTTACCTTTGCTACCTTC	
pBAD-LptD-F	CTAGCAGGAGGAATTCATGAAAAAACGTATCCCCAC	pBAD cloning
pBAD-LptD-R	GCAGGTCGACTCTAGAGGATTACGTGGTAAATCAAC	

pET24a-PssA-F	AAGGAGATATACATATGTTGTCAAAATTTAAGCG	pET cloning
pET24a-PssA-R	GCTCGAATTCGGATCCAATAGCGAACAGACAAAAAG	
pET28a-YgfB-F	CGCGCGGCAGCCATATGTCTATACAGAACGAAAT	
pET28a-YgfB-R	GCTCGAATTCGGATCCACTCATAACACTCTCCTTAC	
pKT25-PssA-F	CGACTCTAGAGGATCCCATGTTGTCAAAATTTAAGCG	Bacterial two-hybrid cloning
pKT25-PssA-R	AACGACGGCCGAATTCAATAGCGAACAGACAAAAAG	
pUT18c-YgfB-F	CGACTCTAGAGGATCCCATGTCTATACAGAACGAAAT	
pUT18c-YgfB-R	TTATATCGATGAATTCACTCATAACACTCTCCTTAC	
pRL27-inner-F1	GGTTGTAACACTGGCAGAGCATTACG	Random mutagenesis
pRL27-SynArb1	CCCACAGGAAGGTATTCTGGAAGATACGGCGGT	
pRL27-inner-F2	ATCAGCAACTTAAATAGCCTCTAAGG	

Table S3. Minimal inhibitory concentrations (MICs) of various antibiotics in indicated cells

Antibiotics \ Strains		WT	*ΔlptD* / pBAD-LptD (0.001% arabinose)	*ΔlptD* / pBAD-LptD (0.01% arabinose)	*ΔlptD* / pBAD-LptD (0.1% arabinose)
β-lactam	Cefalotin	8[a]	0.5	1	8
	Cefoxitin	2	2	4	4
	Imipenem	0.5	0.25	0.25	0.25
Metabolite derivative	Fosfomycin	4	2	2	1
Tetracycline	Minocylcine	1	0.5	0.125	0.25
Lincosamide	Clindamycin	256	8	8	16
Steroide	Fusidic acid	512	256	256	256
Quinolone	Nalidixic acid	4	1	8	2
Ansamycin	Rifampicin	8	0.125	0.5	4
Etc.	Nitrofurantoin	8	0.5	2	8

[a]The MIC (μg/ml) is the lowest antibiotic concentration preventing the lawn growth of the bacteria.

References

1. Blattner FR, Plunkett G, 3rd, Bloch CA, Perna NT, Burland V, Riley M *et al.* 1997. The complete genome sequence of *Escherichia coli* K-12. *Science* **277:** 1453-1462.

2. Park SH, Kim YJ, Lee HB, Seok YJ, Lee CR. 2020. Genetic evidence for distinct functions of peptidoglycan endopeptidases in *Escherichia coli*. *Front Microbiol* **11:** 565767.

3. Karimova G, Pidoux J, Ullmann A, Ladant D. 1998. A bacterial two-hybrid system based on a reconstituted signal transduction pathway. *Proc Natl Acad Sci U S A* **95:** 5752-5756.

4. Datsenko KA, Wanner BL. 2000. One-step inactivation of chromosomal genes in *Escherichia coli* K-12 using PCR products. *Proc Natl Acad Sci USA* **97:** 6640-6645.

5. Larsen RA, Wilson MM, Guss AM, Metcalf WW. 2002. Genetic analysis of pigment biosynthesis in *Xanthobacter autotrophicus* Py2 using a new, highly efficient transposon mutagenesis system that is functional in a wide variety of bacteria. *Arch Microbiol* **178:** 193-201.
